# Rain increases the energy cost of bat flight

**DOI:** 10.1098/rsbl.2011.0313

**Published:** 2011-05-04

**Authors:** Christian C. Voigt, Karin Schneeberger, Silke L. Voigt-Heucke, Daniel Lewanzik

**Affiliations:** 1 Evolutionary Ecology Research Group, Leibniz Institute for Zoo and Wildlife Research, Alfred-Kowalke-Strasse 17, 10315 Berlin, Germany; 2 Animal Behaviour, Freie Universität Berlin, Takustrasse 6, 14195 Berlin, Germany

**Keywords:** aerodynamics, Chiroptera, energetics, flight costs, thermoregulation, vertebrate flight

## Abstract

Similar to insects, birds and pterosaurs, bats have evolved powered flight. But in contrast to other flying taxa, only bats are furry. Here, we asked whether flight is impaired when bat pelage and wing membranes get wet. We studied the metabolism of short flights in *Carollia sowelli*, a bat that is exposed to heavy and frequent rainfall in neotropical rainforests. We expected bats to encounter higher thermoregulatory costs, or to suffer from lowered aerodynamic properties when pelage and wing membranes catch moisture. Therefore, we predicted that wet bats face higher flight costs than dry ones. We quantified the flight metabolism in three treatments: dry bats, wet bats and no rain, wet bats and rain. Dry bats showed metabolic rates predicted by allometry. However, flight metabolism increased twofold when bats were wet, or when they were additionally exposed to rain. We conclude that bats may not avoid rain only because of sensory constraints imposed by raindrops on echolocation, but also because of energetic constraints.

## 1. Introduction

In vertebrates, powered flight has evolved three times, but only Chiroptera are furry and use flexible wing membranes for flapping flight. So far, the aerodynamics and energetics of bat flight have been mainly studied under ideal conditions, such as in controlled laboratory settings and in wind tunnels [1,2]. But it is unknown how flying bats perform when conditions turn suboptimal, such as during rain. Indeed, field observations confirm that bats avoid rain. For example, insectivorous hoary bats (*Lasiurus cinereus*) stop foraging and retreat into the vegetation during heavy rainfall but continue to forage in light rain [3]. Two explanations seem plausible for this behaviour. First, raindrops may interfere with echolocation, making it less easy for bats to detect insect prey or obstacles [4]. Second, perhaps bats avoid rain because the moistening of their body inflicts energy costs on flight by reducing lift and thrust production, or by adding thermoregulatory costs. Indeed, when 0.1 ml of water droplets evaporate from the body surface during a 1 min flight, an 18 g bat has to invest 4 W of thermoregulatory costs in order to maintain normal body temperature [5]. This is about twice the flight cost that the same bat would encounter under dry conditions [2].

Here, we test the idea that rain imposes energy costs on flying bats. We quantified the metabolic rate of short flights in Sowell's short-tailed fruit bat (*Carollia sowelli*). This Central American species encounters frequent and heavy rainfall. We studied flight metabolism using the ^13^C-labelled Na-bicarbonate (NaB) method, modified for bolus injections in flying endotherms [6]. We exposed bats to three treatments in an outdoor flight enclosure. We tested bats flying under (i) dry conditions, (ii) with moistened pelage and wing membranes but without rain, and (iii) as in (ii) but with rain. We predicted that flight metabolism is higher when bats are wet or when they are additionally exposed to rain than when they are dry.

## 2. Material and methods

In 2010, we captured 10 adult *Carollia sowelli* (six males and four females) between 17.00 and 19.00 h, using 6 and 9 m mist nets (2.5 m height, Ecotone, Gdynia, Poland) at La Selva Biological Station in Costa Rica (10°25′ N, 84°00′ W). Individually marked bats were kept in groups of two to four in outdoor flight cages (1 m^3^). Experiments were conducted under the permission of SINAC in Costa Rica and according to the local regulations of the Organization for Tropical Studies. Bats were exposed to three treatments in random order. Animals were allowed to fly without rain, either dry (dry bats) or after moistening their pelage and wing membranes with tap water (wet bats/no rain). Lastly, we exposed wet bats to moderate rainfall (wet bats/with rain). We conducted one trial per night with a given individual. Rain experiments were usually conducted during natural rain. In the absence of rain, we sprayed water above the cage ceiling (wire mesh) with a water hose so that artificial raindrops fell vertically into the flight cage. We measured the amount of water that had accumulated in a bucket set up in the middle of the flight cage. On average, bats experienced 0.88 ± 0.3 l min^−1^ m^−2^ rain during the rain trials, which was similar to a moderate tropical rain (C. C. Voigt 2010, personal observation).

We used the NaB technique as outlined in Hambly *et al*. [6] and modified according to Voigt & Lewanzik [7] for instantaneous measurements of ^13^C enrichments in exhaled breath using a cavity ringdown spectrometer. We performed experiments with one bat at a time. After administering 200 mg isotonic ^13^C-labelled NaB solution (0.29 mol l^−1^; Euriso-Top GmbH, Saarbrücken, Germany) intraperitoneally, we transferred bats into a 1.8 l chamber in which the temperature was kept constant at 30°C (see [7] for a detailed description of the set-up). At about time (*t*) = 12 min post-injection, we transferred bats into a nearby octagonal outdoor flight cage (15.6 m^2^, 2 m height) that was dimly illuminated. After the bats had flown for on average 72.5 ± 8.5 s, we brought them back to the chamber where they stayed for a 10 min post-flight period. Bats were weighed to the nearest 0.01 g using a precision electronic balance (PM-100, Mettler, Switzerland) and transferred back to the maintenance cage. After the experiments, bats were released close to the site of their capture. For data analysis, we focused on a 20 min period about 3 min after peak enrichment in ^13^C. This interval consisted of a pre-flight period (*ca* 5 min), the flight period (*ca* 5 min, including transfers) and the post-flight period (*ca* 10 min). To calculate the fractional turnover of ^13^C (*k*_c_; min^−1^) in flying bats, we converted delta values into atom% [8] and computed linear regressions after the least-squares method for the ln-transformed isotopic data against time for the pre- and post-flight periods separately. These regressions served to extrapolate the ^13^C enrichment in the exhaled breath of animals at the onset and end of the flight trial. The time delay between the end of the pre-flight and onset of flight (start) was *ca* 27 s and the delay between the end of flight (stop) and onset of post-flight period was *ca* 80 s. We calculated *k*_c_ for flying bats according to: *k*_c_ = [*AP*^*13*^*CE*_stop_ – *AP*^*13*^*CE*_start_]/*t*, where *AP*^*13*^*CE* was the ^13^C excess enrichment (in atom%) at the start and stop of the flight trial and *t* the flight duration (min). *k*_c_ (min^−1^) was multiplied by the total body bicarbonate pool *N*_c_ (mol) as calculated by the plateau method [7], and converted to carbon dioxide production rate (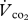; ml min^−1^) by multiplication with 22.4 l mol^−1^. Since previous validation experiments suggested that 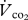 is overestimated when based on *k*_c_ and *N*_c_ (e.g. [6]), we used a correction factor to estimate the 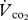 of flying bats. This correction factor was derived from the respirometric and isotopic measurements of the 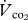 of the pre-flight period. We calculated the *k*_c_ of resting bats using the slope of the pre-flight regression equation. By multiplying *k*_c_ (min^−1^) with *N*_c_ (mol) and 22.4 l mol^−1^, we derived 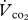 according to the isotopic data, and by multiplying the combined concentrations of ^13^CO_2_ and ^12^CO_2_ (ppm) of the same pre-flight period with the flow-through rate in the chamber, we obtained 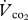 according to the respirometric data [9]. A general linear model with 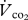 based on isotopic data as the independent variable, 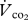 based on respirometry as the dependent variable and individuals as cofactor demonstrated the high precision of this model (multiple *r* = 0.842). We then used the ratio of respirometric and isotopic 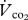 of pre-flight resting bats to calculate the 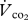 of flying bats based on *k*_c_ and *N*_c_.

We tested for differences in body masses among treatments using repeated measures analysis of variance, and for differences in resting 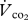 between pre- and post-flight period and among individuals and treatments using a general linear model. We used a Friedman test followed by post hoc Dunn's test to test for differences in 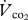 rate among treatments because variances varied greatly among treatments for 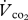 of flying bats. We assumed an alpha value of 5 per cent and used Systat (v. 11). Data are presented as means ± 1 s.d.

## 3. Results

**Figure 1.**
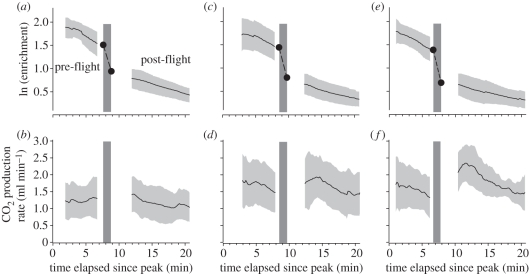
Elimination of ^13^CO_2_ from the body bicarbonate pool (note logarithmic scale) and rate of CO_2_ production (ml min^−1^) in *Carollia sowelli* in relation to time elapsed since peak enrichment ((*a*,*b*) dry; (*c*,*d*) wet + no rain; (*e*,*f*) wet + rain). Solid lines depict means and light grey areas the range of ± one standard deviation. Dashed lines indicate the fractional turnover of flying bats based on extrapolated ^13^C enrichments at the onset and end of the flight period (dark grey rectangle, flight period).

**Figure 2.**
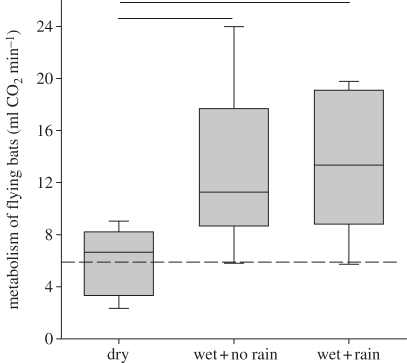
Metabolic rates (ml CO_2_ min^−1^) of flying *Carollia sowelli* when either exposed to dry conditions, wet fur and no rain, or wet fur and rain. Box margins indicate the 25 and 75 percentiles, whiskers the five and 95 percentiles, the centre line of the box the median. Significant differences between treatments are indicated by horizontal lines. The dashed line marks the predicted flight metabolism.

Resting metabolic rates differed among individuals (*F*_9,47_ = 2.51; *p* = 0.020) and treatments (*F*_2,47_ = 6.1, *p* = 0.004; in the electronic supplementary material, table S1), but not between pre- and post-flight periods (*F*_1,47_ = 0.38, *p* = 0.542; figure 1). Following peak enrichments of ^13^C in bat breath after about 7 min, ^13^C enrichment declined steadily in resting *C. sowelli* (figure 1). Bat pelage clumped partly together when we moistened bats with water. But despite this additional load of water, bats did not differ in body mass among treatments (*F*_2,29_ = 135.2, *p* = 0.51). Experimental bats weighed on average 17.7 ± 2.2 g. Flight metabolism of bats differed among treatments (*n* = 10, *k* = 3, *Fr* = 12.7, *p* = 0.0017; figure 1). Metabolic rates of dry bats averaged 6.1 ± 2.5 ml CO_2_ min^−1^, which did not deviate from the predicted value of 6.0 ml CO_2_ min^−1^ for a 17.7 g bat ([2]; Student *t*-test, *t*_9_ = 0.39, *p* = 0.702). Wet bats encountered higher flight metabolic rates than dry bats (no rain: 12.9 ± 6.0 ml CO_2_ min^−1^; mean rank difference = 12.5, *p* < 0.01; with rain: 13.6 ± 5.4 ml CO_2_ min^−1^; mean rank difference = 11.8, *p* < 0.01; figure 2). Exposure to the rain did not alter wet bats' metabolic rates (mean rank difference = 0.7; *p* > 0.05).

## 4. Discussion

Bats exhibited a higher flight metabolism with wet fur than with dry fur. Since exposure to rain did not add surplus energy costs for flying bats, we infer that the moistening of the pelage and wing membranes was associated with the increased metabolic rate and not, for example, an altered flight behaviour caused by falling raindrops. Theoretically, flight costs should increase to some extent because water trapped in the pelage adds mass to bats. However, a twofold increase in flight costs would involve an additional water load of 25 g for an 18 g bat [2], which seems to be an unlikely scenario. Possibly, we could not detect any difference in body mass between dry and wet bats because the amount of water trapped in the pelage was negligible in relation to the large variation in body mass between days. The cooling effect of water evaporating from the body surface of flying bats could add thermoregulatory costs to flight metabolism. A difference of approximately 7.5 ml CO_2_ min^−1^ in flight metabolism between dry and wet bats translates into 2.1 W, when assuming carbohydrate oxidation. An additional metabolic rate of 2.1 W may compensate for the evaporative cooling effect of 0.05 g H_2_O, an amount of evaporative water loss that seems possible for an 18 g bat flying for a 1 min period. However, we cannot prove unambiguously that the elevated flight costs of wet bats are solely caused by increased thermoregulatory costs, since we lack detailed measurements of evaporative water loss in our study animals. Indeed, high humidity during rain may lower the rate of evaporation and, consequently, the cooling effect [10]. Alternatively, lift and thrust production may change when wet bats increase flight speed or when aerodynamic properties of pelage and wing membranes suffer. This could also inflict energy costs on the flight of wet bats.

Increased flight metabolism of wet bats may explain why bats reduce or cease foraging activities in rain. Bats may only continue to forage in rain when resources offer sufficient energy gain. For example, we observed *Noctilio albiventris* hunting swarming insects at a streetlight even in rain [11], and fruit-eating bats are known to forage in drizzling and moderate rain [12]. Sensory constraints may present an additional problem for echolocating bats when flying in rain, but bats may rather reduce flight activity because of overly high foraging costs when pelage and wing membranes become wet, and not because they lose orientation or the ability to detect prey.
